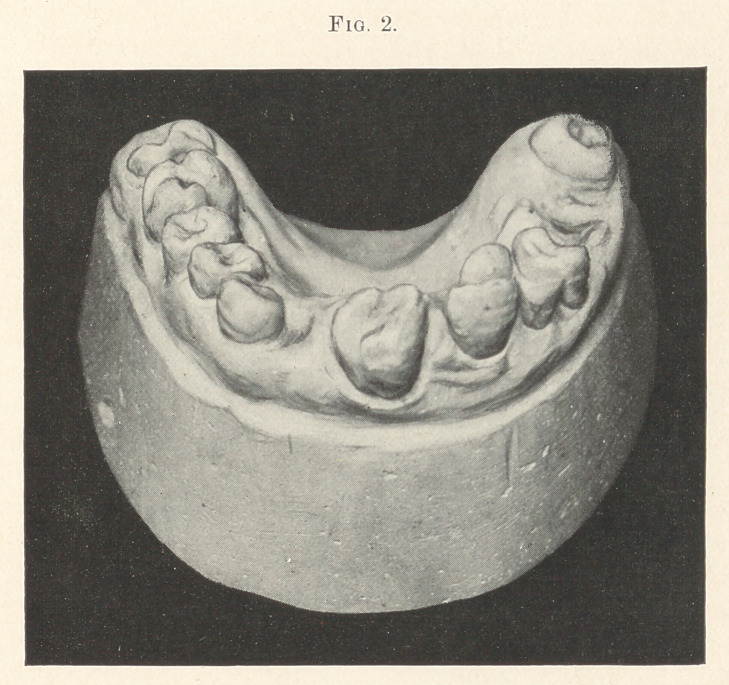# Studies in Evolution

**Published:** 1898-04

**Authors:** Eugene S. Talbot

**Affiliations:** Chicago, Ill.


### STUDIES IN EVOLUTION.

BY EUGENE S. TALBOT, M.D., D.D.S., CHICAGO, ILL.

No tissues of the body are exempt from malformations, but this is more markedly apt to affect structures and organs which are undergoing constant changes during evolution. The face, jaws, teeth, and vermiform appendix are marked examples. In the INTERNATIONAL DENTAL JOURNAL, February, March, and April, 1897, in an article on "The Degenerate Jaws and Teeth," I showed how, in the order of evolution, these structures were degenerating and producing deformities and irregularities of the structures. My results confirmed the concrescence theory of Magitot and the differentiation theory of Osborn and Cope as the manner of formation of the bicuspids and molars.

The lower marsupials and the lemurs had forty-four teeth, and with rise in the scale to man the jaws have become shorter with fewer teeth. As the jaws are still growing smaller, nature is trying to compensate for this by dropping the teeth at the two extremes of the jaw, the laterals and the third molars. The same is true in atavism: the extra teeth in the majority are located in the anterior and posterior part of the jaw. It would be reasonable, therefore, that, under certain conditions, instead of removing the teeth in the anterior and posterior part of the mouth, nature would occasionally produce such freaks as occur in the face, jaws, vermiform appendix, and monstrosities. This is most often the case in the third molar. It is not so frequently the case with the teeth in the anterior part of the mouth.

I have occasionally noticed such cases, two of which are illustrated in this article. These models were obtained at the Dental School in Paris, France, some years ago. Fig. 1 shows all the anterior teeth normal, except the central incisors. Here nature undertook by the differentiation or budding process to produce two molar teeth, but fell far short of it. The right monstrosity is apparently an incisor reversed, the palatine surface being near the lip with a

Fig. 1.

Fig. 2.

number of buds coalescing in the palatine surface. The left monstrosity is more perfectly developed. It is round, with ten or twelve buds situated in different parts of the crown joined together.

Fig. 2 shows a perfect piece of mechanism. In place of the lateral incisor is a molar tooth with cusps and separate roots recognized by the depression at the bifurcation at the gingival margin. The molar has pushed the right central and lateral to the right, causing the cuspid to erupt in the vault. The cusps are very sharp and the sulci deep in the bicuspids and molars, thus showing an imperfect development throughout. These freaks of nature are the result of some disturbances in the development of tissue as early as the third or forth week of fetal life. It may be due to direct inheritance or to idiopathic influential elements of one or both parents.

## Figures and Tables

**Fig. 1. f1:**
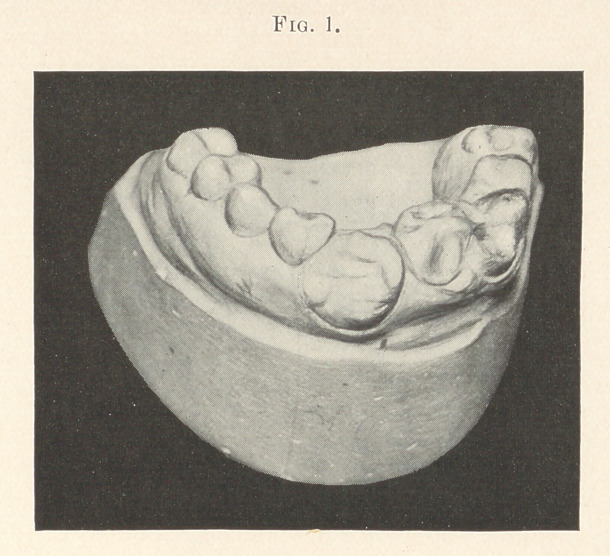


**Fig. 2. f2:**